# Anesthetic Management of a Patient With Post‐Intubation Tracheal Stenosis: Use of V‐V ECMO

**DOI:** 10.1002/ccr3.71905

**Published:** 2026-04-06

**Authors:** Xiaoping Chen, Wei Xie, Ju Gao, Yali Ge

**Affiliations:** ^1^ Clinical Medical College Yangzhou University Yangzhou Jiangsu China; ^2^ Department of Anesthesiology, Institute of Anesthesia, Emergency and Critical Care Northern Jiangsu People's Hospital Affiliated to Yangzhou University Yangzhou Jiangsu China

**Keywords:** induction of general anesthesia, post‐intubation tracheal stenosis, tracheal stenosis, veno‐venous extracorporeal membrane oxygenation

## Abstract

A 34‐year‐old woman with severe post‐intubation tracheal stenosis underwent successful tracheal resection under V‐V ECMO, highlighting its utility in high‐risk airway obstruction. Multidisciplinary teamwork and staged anesthetic protocols further improved peri‐operative safety and patient outcomes.

## 1 Introduction

Tracheal stenosis (TS) is a potentially life‐threatening disorder caused by post‐intubation injury, infection, inflammation, or idiopathic factors. Post‐intubation tracheal stenosis (PITS) represents the most frequent form of acquired benign stenosis [1]. PITS usually results from excessive cuff pressure or prolonged intubation (> 7 days); ensuing mucosal ischemia promotes fibrosis or granulation‐tissue formation [1]. Severity assessment should integrate luminal diameter, lesion histology, and clinical presentation. A composite scoring system quantifies stenosis by lumen narrowing, histopathology, and respiratory impairment; a total score ≥ 8.5 indicates surgical treatment [2]. Peri‐operative anesthetic management of these patients is therefore particularly demanding. Induction of general anesthesia risks airway collapse or failed intubation, and intra‐operative ventilation can be inadequate, causing hypoxaemia and haemodynamic instability. Consequently, conventional airway strategies often prove insufficient for such high‐risk procedures.

Extracorporeal membrane oxygenation (ECMO) is an established extracorporeal life‐support modality that offers a novel solution for high‐risk airway surgery.

By oxygenating blood and removing carbon dioxide without endotracheal intubation, ECMO eliminates mechanical trauma to a narrowed airway and provides a stable operative field. Reports describe successful ECMO use during tracheal tumor excision, airway stenting, and complex tracheal reconstruction, markedly enhancing procedural safety [3, 4]. Veno‐venous (V‐V) ECMO is preferred in patients with preserved cardiac function because it minimally disturbs haemodynamics [4].

We present a patient with severe PITS who underwent emergency segmental tracheal resection under V‐V ECMO, general anesthesia, and multidisciplinary guidance. This experience underscores ECMO's pivotal role in high‐risk airway surgery and may inform peri‐operative management of similarly complex cases.

## 2 Case History

**FIGURE 1:**
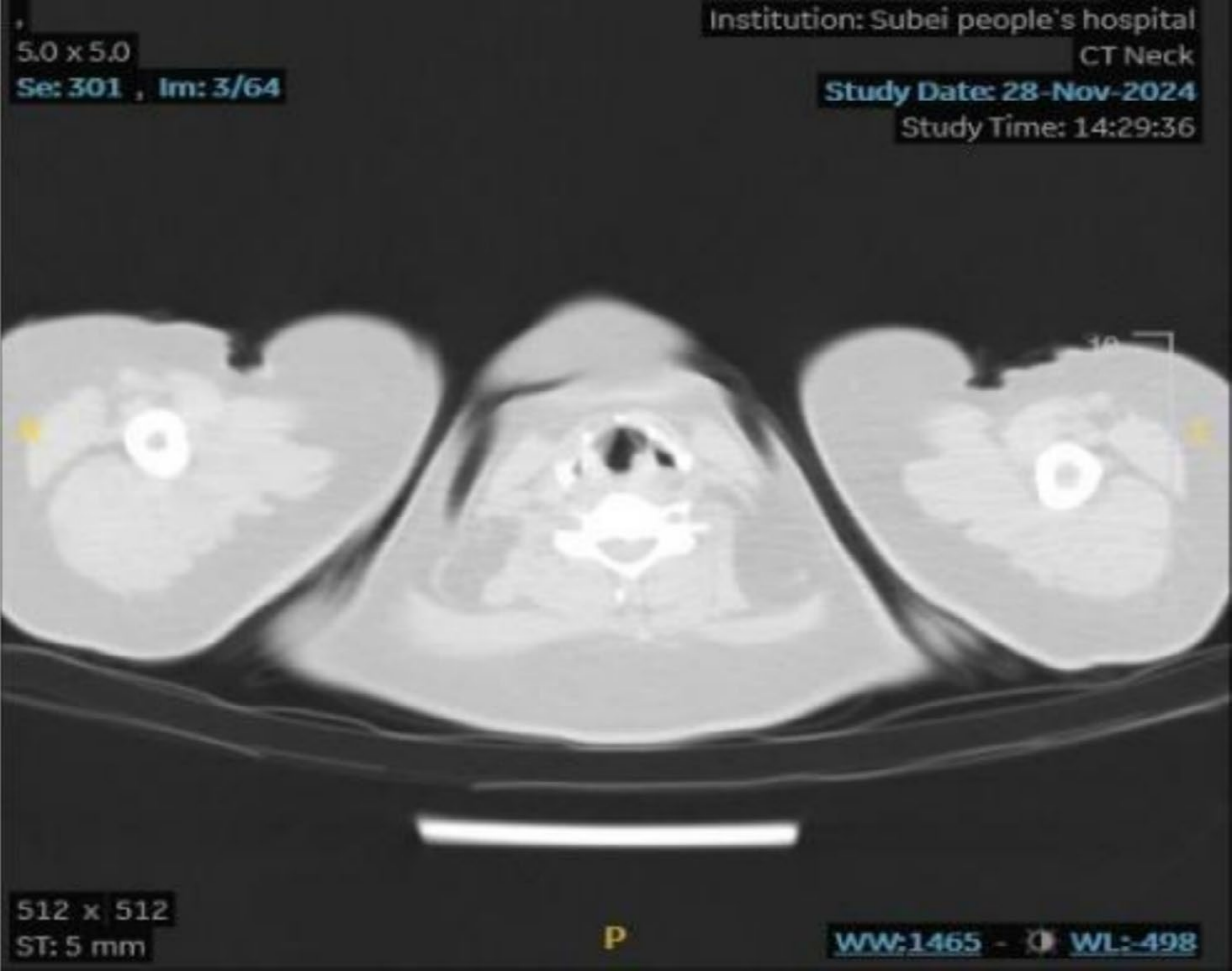
Preoperative neck CT scan.

A 34‐year‐old woman was admitted with an 8‐day history of wheezing. She underwent endotracheal intubation for diabetic ketoacidosis in the ICU of Northern Jiangsu People's Hospital on 27 October 2024 and was discharged on 11 November after clinical improvement and extubation. She presented to our hospital with chest tightness, shortness of breath, and inspiratory dyspnoea. Cervical CT revealed focal tracheal narrowing at the T2 level (Figure 1). Laboratory tests showed leukocytes 10.44 × 10^9^/L, neutrophils 7.29 × 10^9^/L, random blood glucose 7.1 mmol/L, and calcium 2.58 mmol/L; troponin and D‐dimer were within normal limits. Empirical therapy with aminophylline, methylprednisolone sodium succinate, and ceftazidime was initiated, and a provisional diagnosis of tracheal stenosis was established.

**FIGURE 2:**
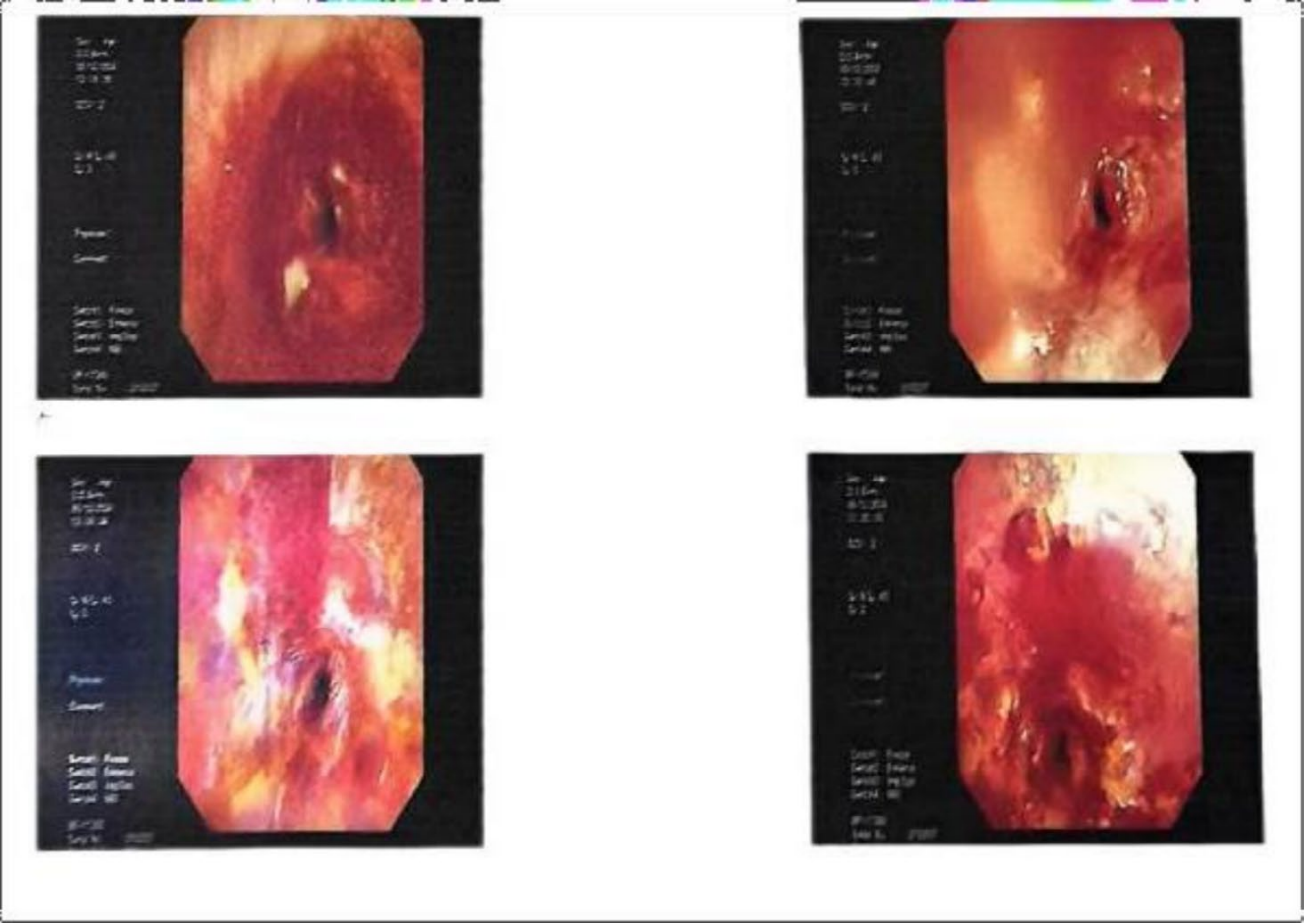
Bronchoscopy of upper trachea.

Type 2 diabetes mellitus had been diagnosed 1 month earlier; glycaemic control consisted of metformin 500 mg twice daily and gliclazide 30 mg once daily. Arterial blood gas analysis on 100% oxygen showed pH 7.27, SaO_2_ 99%, PaO_2_ 167.2 mmHg, and PaCO_2_ 64.2 mmHg. Flexible tracheoscopy revealed mucositis, scarring, and critical narrowing of the upper trachea (Figure 2). Working diagnoses were severe tracheal stenosis and type 2 diabetes mellitus. She remained in a semi‐sitting posture because of marked dyspnoea and inspiratory difficulty. Following multidisciplinary discussion among respiratory physicians, thoracic surgeons, anesthetists, and intensivists, emergency segmental tracheal resection under general anesthesia with ECMO support was planned.

### 2.1 Treatment

The conscious but agitated patient was moved on a flat trolley to the operating theater in a semi‐recumbent position while receiving transnasal high‐flow oxygen at 30 L/min (FiO_2_ 1.0); marked dyspnoea and stridor persisted. Continuous ECG monitoring was instituted immediately: NIBP 175/100 mmHg, HR 135 bpm, SpO_2_ 95%. After routine checks, an ultrasound‐guided cannula was placed in the right dorsalis pedis artery for invasive pressure monitoring and another in the left femoral vein for venous access, both under local anesthesia. Dexmedetomidine was infused via the left femoral line: loading 0.6 μg/kg over 10 min, then 0.2–0.5 μg/kg/h; heart rate was permitted to decrease to ~90 bpm.

Veno‐venous ECMO was established under local anesthesia. A 23 Fr drainage cannula was inserted into the right femoral vein under ultrasound guidance, and a 19 Fr return cannula was placed in the right internal jugular vein. The ECMO circuit was primed and systemic heparinization was achieved with an initial bolus of 100 IU/kg of heparin, followed by a continuous infusion at a rate of 10–15 IU/kg/h to maintain an activated clotting time (ACT) between 180 and 220 s. Once initiated, the ECMO pump flow was titrated to a target range of 2.8–3.2 L/min (approximately 50–60 mL/kg/min), with the rotational speed (RPM) adjusted accordingly to achieve this flow. The sweep gas (100% oxygen) flow was initially set at 2.5 L/min and adjusted based on serial arterial blood gas (ABG) results to maintain a normal PaCO_2_ (35–45 mmHg) and adequate oxygenation (PaO_2_ > 80 mmHg). Intraoperative monitoring included continuous invasive arterial pressure, central venous pressure, pulse oximetry, and frequent ABG analysis.

The circuit flow was titrated to target, enabling discontinuation of high‐flow nasal oxygen while preserving adequate oxygen delivery. Once haemodynamic and oxygenation parameters stabilized, induction was achieved with propofol 70 mg, sufentanil 10 μg, and rocuronium 60 mg through the femoral line. Anesthesia was maintained with propofol and remifentanil infusions, supplemented by intermittent rocuronium boluses. Bispectral index remained between 40 and 60 throughout.

**FIGURE 3:**
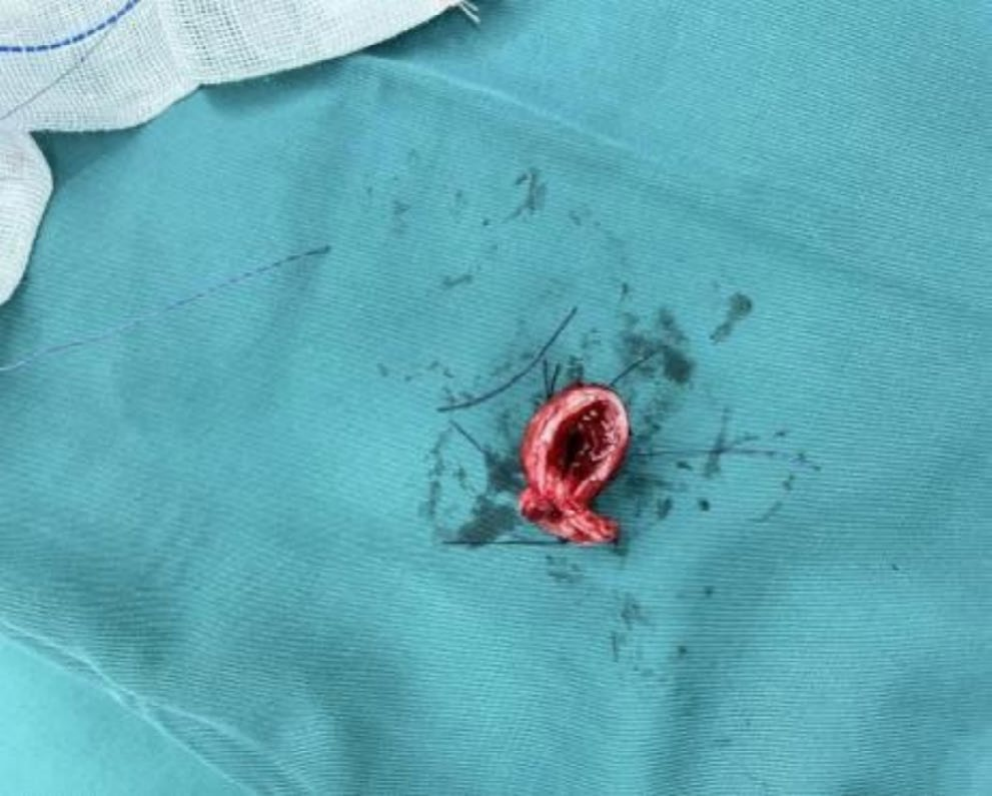
Surgical specimen of tracheal mass.

Under bronchoscopic guidance the trachea was divided proximal and distal to the lesion. Circumferential granulation tissue formed a web, reducing the lumen to ≈0.2 cm. The 1.5 × 1.0 cm stenotic segment was excised (Figure 3), giving a total resection length of ≈2 cm. The membranous wall was closed with a continuous suture, which was secured with a single knot after tensioning. At closure the chin was sutured to the anterior chest wall (Pearson position) to reduce anastomotic tension. She was transferred to the ICU on ECMO with stable haemodynamics.

### 2.2 Outcome and Follow‐Up

Post‐operative day 2: ECMO discontinued and high‐flow nasal oxygen commenced. Post‐operative day 3: oxygen reduced to a standard nasal cannula. With stable observations and no respiratory distress, she was stepped down to the thoracic ward on day 5. No immediate complications, such as significant bleeding, cannula displacement, or limb ischemia, were encountered during the perioperative period. Distal limb perfusion of the cannulated right leg was monitored clinically and by Doppler ultrasound every 4 h, and remained satisfactory throughout. The patient did not develop any signs of catheter‐related bloodstream infection during or after the ECMO run. Finally, she was discharged home in good condition.

**FIGURE 4:**
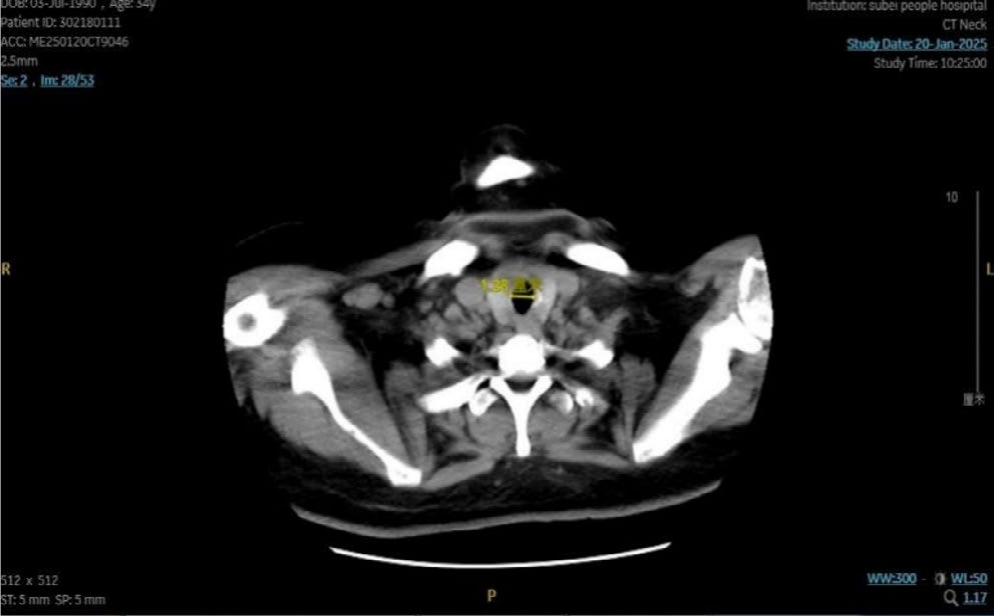
Postoperative neck CT scan.

One month after the operation, the patient was followed up. The patient was in good mental state, without breathing difficulties, coughing or expectoration. The incision of the neck surgery recovered well. The result of the neck CT examination showed that the trachea was in the middle, no obvious stenosis was seen in the lumen, and the airway was unobstructed (Figure 4).

## 3 Discussion

Airway stenosis often results from benign or malignant tracheal disease. The etiology can be grouped into three categories [5]: (1) intraluminal pathology—benign or malignant tracheobronchial tumors and tuberculous granulomas; (2) extrinsic compression by neighboring masses such as esophageal carcinoma, thyroid or mediastinal tumors; and (3) airway‐wall disorders including tracheomalacia, post‐tracheotomy sequelae, or intubation‐related granuloma. The present case represents post‐intubation tracheal stenosis (PITS) caused by extended intubation and excessive cuff pressure [1]. She became comatose from diabetic ketoacidosis and required ICU intubation for > 10 days. Cuff pressure was not monitored continuously during that time. Histology showed focal epithelial denudation with exuberant granulation tissue, marked fibrosis, and dense inflammatory infiltrates. These changes confirmed the diagnosis of PITS.

Management is tailored to history, imaging/endoscopic findings, and cardiopulmonary reserve. In mild–moderate disease, endoscopic measures—balloon dilation, stenting, or laser ablation—are first‐line. For severe or recurrent stenosis, segmental resection with primary end‐to‐end anastomosis remains the gold standard, relieving symptoms and improving quality of life in ≈90% of patients [6].

Tracheal surgery is intrinsically complex and demands a multidisciplinary team of surgeons, anesthetists, intensivists, and specialist nurses. For anesthetists, the principal challenge is airway management. Meticulous pre‐operative assessment and an individualized anesthetic plan are paramount. The procedure can be divided into five key stages: (1) induction and airway establishment; (2) lesion localisation; (3) operative‐field intubation and ventilation; (4) tracheal anastomosis; and (5) emergence and extubation. Stages (1) and (5) carry the greatest anesthetic risk—initial airway control and the return to spontaneous ventilation [7].

Management must be tailored to patient‐specific factors. Reported airway techniques include conventional intubation, tubeless approaches, laryngeal mask, high‐frequency jet ventilation, cardiopulmonary bypass, and ECMO [8, 9]. No randomized trials directly compare these methods, so none can be deemed superior. We advocate a structured pre‐operative evaluation that integrates history, imaging, bronchoscopy, and physiology: (1) Lesion location—distance from glottis and carina; (2) lesion size—degree of obstruction, positional variability, estimated rings to resect; (3) lesion characteristics—benign vs. malignant, consistency, friability; (4) airway difficulty—anticipated mask ventilation and intubation challenges; (5) co‐morbidities—cardiopulmonary reserve and hypoxia tolerance.

**FIGURE 5:**
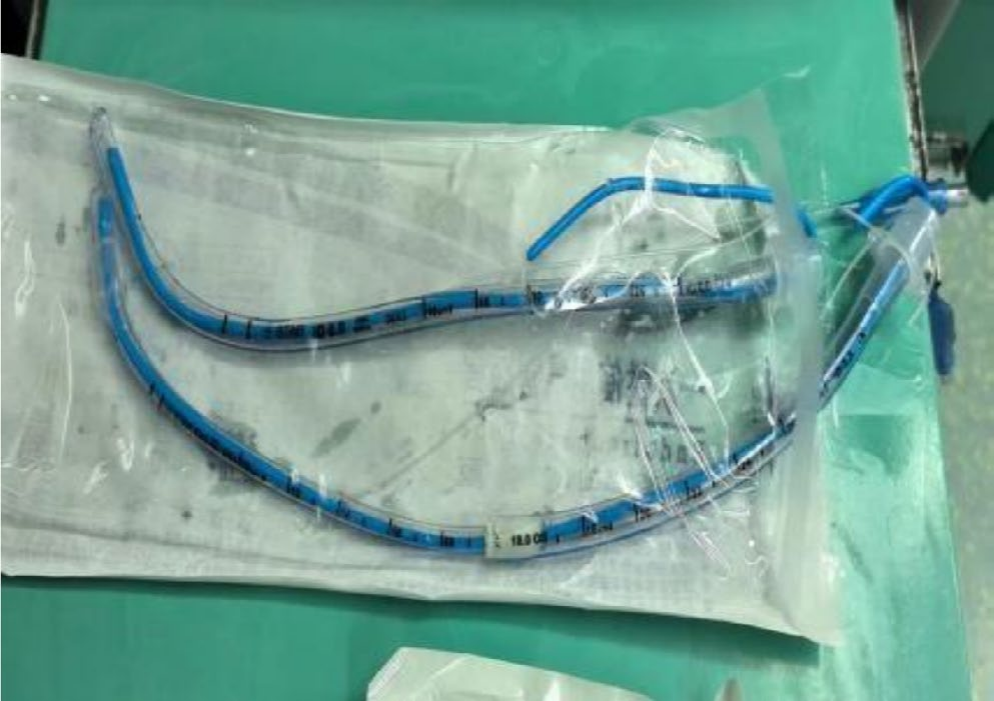
Self‐made emergency long tracheal catheter.

Pre‐operatively the patient had critical subglottic stenosis: ≈90% luminal narrowing over a 1.5 cm segment situated 5 cm above the carina, through which a fiber‐optic bronchoscope could not be advanced. She was agitated and could breathe only while sitting upright, so resection under preserved spontaneous ventilation was unrealistic. Induction of general anesthesia risked total airway collapse, a difficult or traumatic intubation, and further damage; conventional intubation was therefore contraindicated. Because the stenosis resulted from intubation‐induced granulation tissue, a no‐intubation strategy was adopted. A five‐step peri‐operative airway plan was formulated after multidisciplinary discussion. (1) Maintain oxygenation with high‐flow nasal cannula; have a custom long anterior endotracheal tube (Figure 5) ready for emergency use. (2) Initiate bedside veno‐venous ECMO and taper high‐flow oxygen once adequate flow is achieved. (3) After tumor excision, perform airway toileting under transnasal bronchoscopy; the surgeon simultaneously suctions with a sterile catheter. (4) On completion, transfer the patient to ICU on ECMO without tracheal intubation. (5) Discontinue ECMO once spontaneous ventilation is adequate. Given the severe scar‐related stenosis, ECMO provided the safest means of completing the procedure and maximizing patient benefit.

Extracorporeal membrane oxygenation (ECMO) is an advanced life‐support modality that has proved valuable both for critical airway obstruction during tracheal surgery [3] and for complex thoracic tumor resection requiring cardiopulmonary assistance [4]. By providing dependable cardiopulmonary support, ECMO is now the technique of choice for severe tracheal stenosis and extensive tumor resection. It affords optimal exposure and ample operative time. Whether to intubate and wean ECMO in theater must be decided on a case‐by‐case basis. After multidisciplinary review, the team agreed that prior intubation had precipitated the stenosis and should be avoided. Further, re‐intubation risked anastomotic trauma. Operating without a tube enabled the surgeon to inspect the suture line for bleeding or leakage, a judgment the anesthetist could corroborate with a fiber‐optic bronchoscope. Safe timing of ECMO weaning was therefore pivotal. A planned period of postoperative observation on ECMO allowed the patient to traverse the high‐risk window safely. Potential drawbacks of ECMO—higher cost, bleeding, catheter‐related infection, and limb ischaemia—must, however, be weighed. In our case, the activated clotting time was closely monitored and maintained within the therapeutic range (180–220 s) to balance anticoagulation and bleeding risk. Strict aseptic techniques were employed during cannulation. Distal limb perfusion was assessed regularly by clinical examination and, if needed, Doppler ultrasound. Fortunately, the patient did not experience any ECMO‐related complications, and the circuit was successfully weaned 8 h postoperatively. With vigilant monitoring and a proficient multidisciplinary team, the risks of ECMO can be effectively mitigated in a controlled surgical setting, thus making it a highly advantageous option for severe tracheal stenosis.

Management of severe tracheal stenosis varies according to etiology and anatomical location. Table 1 compares the anesthetic protocols for the index case with two previously treated patients who had representative airway lesions. Case 2 involved critical stenosis (≈90% obstruction) from a tracheal tumor located 1.5 cm below the glottis. Induction was undertaken without tracheal intubation. High‐flow nasal oxygen extended the safe apnoea time, while ultrasound‐guided cervical nerve block with topical anesthesia blunted the stress response, enabling the patient to tolerate tracheotomy. After tracheotomy, distal tracheal intubation was placed and surgery proceeded uneventfully. The index patient differed from Case 2 in both etiology—intubation‐induced scarring—and location, with stenosis near the carina. A total “no‐tube” strategy therefore avoided further irritation and optimized the operative field.

**TABLE 1:** Clinical characteristics and airway management of three cases with tracheal stenosis.

Parameter	Case 1	Case 2	Case 3
Sex	Female	Female	Female
Age (years)	34	54	39
BMI	25.4	24	20.3
ASA	IV	III	II
Degree of tracheal obstruction (%)	90	90	50
Distance from upper margin of mass to glottis (cm)	6	1.5	3.5
Distance from lower margin of mass to carina of trachea (cm)	5	7	8
Etiology of stenosis	Post‐intubation scar and granulation	Adenoid cystic carcinoma	Adenoid cystic carcinoma
Position	Semi‐Fowler's position	Semi‐recumbent position	Supine position
Dyspnea	Present	Exertional dyspnea	Mild exertional dyspnea
Resting stridor	Present	Present	None
Respiratory failure	Type II	None	None
Comorbidities	Diabetes mellitus	None	None
Airway management	V‐V ECMO	Non‐intubation	Endotracheal intubation

Abbreviations: ASA, American Society of Anaesthesiologists; BMI, body mass index; cm, centimeter; V‐V ECMO, veno‐venous extracorporeal membrane oxygenation.

Case 3 showed moderate (≈50%) tracheal narrowing produced by a tumor at the T1 level, 3.5 cm below the glottis. Pre‐operative CT indicated that a 6.5 mm ID tube could be accommodated and the patient was asymptomatic, yet care was required to avoid tumor contact; therefore a conventional airway plan was selected. Intubation was performed under fiber‐optic guidance to ensure the distal tip remained above the tumor's upper pole. Thorough pre‐oxygenation with high‐flow mask or nasal cannula is essential to extend the safe apnoea window during induction. For mild–moderate stenosis, evidence suggests that an obstruction ≤ 75% with a residual lumen ≥ 5 mm usually permits tracheal intubation [5]. Nevertheless, manipulation of a tracheal tumor risks rupture and bleeding, so fiber‐optic guidance offers an added safety margin.

Literature indicates that a laryngeal mask airway (LMA) minimizes airway manipulation, thereby reducing intubation‐related bleeding or mucosal trauma and improving patient comfort at emergence. Recommended indications for LMA in tracheal surgery are strict: the lesion must lie ≥ 2 cm below the glottis, residual lumen ≥ 65% (< 35% obstruction), and the pathology should be benign scar‐related stenosis [10]. In Case 2 the LMA was rejected because neck extension for the operative field could displace the device and cause hypoventilation; moreover, a distal tracheal tube was required at closure to let the surgeon check the anastomosis for bleeding or air leak. Furthermore, the high‐grade stenoses in Cases 1 and 3 rendered LMA use unsafe.

High‐frequency jet ventilation (HFJV) is well suited to open tracheal surgery because it preserves oxygenation yet leaves the surgical field unobstructed [11]. Before the trachea is opened, however, HFJV can markedly increase the risk of CO_2_ retention, air trapping, and barotrauma—particularly in severe stenosis. Accordingly, clinicians confine HFJV to the interval between tracheotomy and completion of the anastomosis. During prolonged jet ventilation, arterial blood gases must be checked frequently to monitor PaCO_2_ and avert postoperative complications [8]. Although none of the three patients required HFJV, anesthetists should still master this key technique for emergencies.

Therefore, compared with non‐ECMO strategies such as HFJV or LMA, V‐V ECMO offers distinct advantages in cases of near‐total tracheal obstruction where conventional ventilation is unfeasible. LMA, though less invasive, is unsuitable for high‐grade stenoses or lesions close to the glottis due to risks of displacement and inadequate ventilation. While HFJV can provide adequate gas exchange during open tracheal surgery, it carries risks of barotrauma, air trapping, and hypercapnia in the setting of severe stenosis, as the high‐pressure jet may not adequately vent. In our patient (Case 1), the “no‐tube” strategy under ECMO not only avoided further airway injury but also provided an unobstructed and stable surgical field, which was critical for precise dissection and anastomosis.

In summary, tracheal resection and reconstruction pose formidable anesthetic challenges. Nevertheless, multidisciplinary teamwork, meticulous pre‐operative assessment of the stenosis, surgical mastery, and an individualized ventilation plan—endorsed by informed patient consent—can together secure a successful outcome.

## Author Contributions

**Xiaoping Chen:** data curation, investigation, writing – original draft. **Wei Xie:** data curation. **Ju Gao:** funding acquisition, methodology, supervision. **Yali Ge:** data curation, investigation.

## Funding

This work was supported by National Natural Science Foundation of China, 82172190.

## Ethics Statement

The authors have nothing to report.

## Consent

Informed consent was obtained from the participant included in the case report. Participant signed informed consent regarding publishing their data.

## Conflicts of Interest

The authors declare no conflicts of interest.

## Data Availability

All data generated during this study are included in this published article.
